# Physiotherapy in Mental Health: A Mixed Methods Systematic Review of Practitioner and Student Knowledge, Attitudes, and Perceptions

**DOI:** 10.1002/pri.70329

**Published:** 2026-08-24

**Authors:** Farahdina Bachtiar, Chiara Mastrogiovanni, Grace McKeon, Davy Vancampfort, Holly Seale, Dicky C. Pelupessy, Afsana Anwar, Hilmi Zadah Faidullah, Brendon Stubbs, Simon Rosenbaum

**Affiliations:** ^1^ Nutrition, Exercise & Social Equity (NExuS) Research Group, Discipline of Psychiatry and Mental Health, School of Clinical Medicine University of New South Wales Sydney Australia; ^2^ Department of Physiotherapy Universitas Pembangunan Nasional Veteran Jakarta Depok Jakarta Indonesia; ^3^ Institute of Physical Activity and Nutrition (IPAN), School of Exercise and Nutrition Sciences Deakin University Geelong Victoria Australia; ^4^ Department of Rehabilitation Sciences KU Leuven Leuven Belgium; ^5^ University Psychiatric Center KU Leuven Leuven Belgium; ^6^ School of Population Health, Faculty of Medicine and Health University of New South Wales Sydney Australia; ^7^ Faculty of Psychology Universitas Indonesia Kampus UI Depok Jawa Barat Indonesia; ^8^ Universitas 'Aisyiyah Yogyakarta Sleman Regency DI Yogyakarta Indonesia; ^9^ Institute of Psychiatry, Psychology, and Neuroscience King's College London London UK; ^10^ Department of Sport and Human Movement Science Centre for Sport Science and University Sports, University of Vienna Wien Austria

**Keywords:** attitudes, knowledge, mental health, physiotherapy

## Abstract

**Background and Purpose:**

Mental health physiotherapy is increasingly recognised as a specialised area of practice. Adequate knowledge and positive attitudes are essential for ensuring safe evidence‐based care and for directly influencing the quality of services delivered. Despite this, research on mental health literacy and attitudes within physiotherapy remains fragmented. This study aims to synthesise evidence on the knowledge, attitudes and perceptions of physiotherapists and physiotherapy students regarding roles in mental health practice.

**Methods:**

A mixed‐methods systematic review was conducted and registered in PROSPERO (CRD42024611691). An initial search of Embase (via Ovid), PubMed, PsycINFO, Scopus, CINAHL and Google Scholar was performed from inception to November 2024, followed by an updated search of the same databases in January 2026. Empirical studies examining knowledge, attitudes or perceptions related to mental health were included. Study quality was appraised using the mixed method appraisal tool (MMAT). Findings were synthesised narratively for quantitative data and thematically for qualitative data, with integration through a convergent segregated approach.

**Results:**

A total of 42 studies were included, comprising 2780 physiotherapists and 2715 students. Participants generally demonstrated positive attitudes towards people with mental illness, whereas reported mental health knowledge varied from limited to good across studies and populations. Experiential learning through direct client interaction appeared to be associated with a more integrated understanding of mental health in physiotherapy and greater confidence, comfort, empathy and reflective capacity. However, the included studies were methodologically heterogeneous, and psychometric properties of the assessment instruments were often insufficiently documented.

**Discussion:**

Participants recognised the importance of mental health support and held positive attitudes, but may not have been prepared to address mental health concerns in physiotherapy practice. Limited education, clinical exposure and role clarity may have contributed to this lack of readiness. These findings support the consideration of more targeted mental health education and structured training initiatives.

**Trial Registration:**

The protocol for this review was registered in the PROSPERO database (CRD42024611691)

## 1 Introduction

Physiotherapists have traditionally focused on physical impairments and functional limitations. However, contemporary healthcare models increasingly call upon the profession to engage more actively in the promotion and support of mental health, recognising the interaction between physical and psychological factors in shaping health outcomes. Since the International Organisation of Physiotherapy in Mental Health (IOPTMH) was established in 2011, the integration of physiotherapy into mental health services has accelerated worldwide (World Physiotherapy 2025). While mental health physiotherapy is recognised as a specialist area, supporting mental health and well‐being is a core responsibility for all physiotherapists. Thus, physiotherapists' preparedness to engage with mental health issues has become an important consideration for both clinical practice and professional education.

Knowledge, attitudes, and perceptions may influence how physiotherapists engage with and respond to individuals experiencing mental health concerns. These factors could affect patient interactions and care experiences (Riffel and Chen 2020). However, knowledge does not necessarily translate into practice, while measured attitudes may not fully reflect clinical preparedness. Examining these constructs together helps provide insight into how physiotherapists' knowledge, attitudes, and perceptions relate to their preparedness to address mental health needs. This is particularly relevant because physiotherapists encounter mental health needs across diverse settings and populations (S. E. Heywood et al. 2022). Experiential learning and exposure to individuals with mental health conditions may support competence and help bridge these gaps by linking knowledge and attitudes with clinical application (Edgar and Connaughton 2021; Happell et al. 2019).

Despite increasing research in this area, evidence on physiotherapists remains fragmented, context‐specific, and inconsistent across regions and practice settings (Alotaibi et al. 2024; Gunduza et al. 2023). Existing reviews have explored selected aspects of this field, including mental health education, physiotherapists' experiences in mental health settings, and the use of psychological interventions (Driver et al. 2017; S. E. Heywood et al. 2022; Hooblaul et al. 2023). However, a comprehensive synthesis integrating quantitative and qualitative evidence on knowledge, attitudes, and perceptions is lacking. Consequently, the available evidence provides an incomplete picture of physiotherapists' and physiotherapy students' perspectives towards mental health and psychiatry.

Additionally, studies in this field have used a range of assessment approaches and measurement instruments. This methodological diversity may limit comparability across studies and underscores the need for a comprehensive synthesis of the evidence. Therefore, this mixed‐methods systematic review aimed to synthesise evidence on physiotherapists' and physiotherapy students' knowledge, attitudes, and perceived professional roles, while also examining the assessment methods and psychometric measures used.

## 2 Methods

This review followed the PRISMA guidelines for systematic reviews and meta‐analyses. The research protocol was registered with the PROSPERO database (CRD42024611691). The PRISMA checklist is provided in Table S1.

### 2.1 Eligibility Criteria

Studies were included if they: (1) involved physiotherapists (any speciality, any country) and physiotherapy students (any country, any university); (2) explored knowledge, attitudes, or perceptions related to providing physiotherapy services to individuals with mental illness; (3) used quantitative, qualitative, or mixed‐methods designs (if data can be clearly extracted from both the quantitative and qualitative components); and (4) were primary studies published in English in peer‐reviewed journals, with no date restrictions. Studies were excluded if they: (1) involved other allied health professionals without separately extractable data for physiotherapists or students; (2) were unpublished works, such as conference abstracts; or (3) were review articles, editorials, or commentaries.

In this review, knowledge refers to the information and level of understanding individuals possess regarding a specific topic, developed through study or experience. Attitudes refer to individuals' feelings or opinions towards a person, issue, or situation that may influence their behaviour or responses. Perceptions refer to beliefs or opinions about an issue based on how situations appear (Cambridge University 2025). Studies using the terms confidence and beliefs were also included. Given variation in terminology, conceptually related constructs were grouped into broader categories for synthesis. Confidence was classified with knowledge‐related constructs because it reflected perceived ability, competence, or preparedness to apply mental health knowledge and skills in practice. Beliefs were classified within the attitude domain, as attitudes are theorised to arise from underlying beliefs about an object, issue, or behaviour (Ajzen 1991). These groupings were used to facilitate synthesis and do not imply conceptual equivalence.

### 2.2 Study Selection

Electronic searches were conducted in Embase (via OVID), PubMed, PsycINFO, Scopus, CINAHL, and Google Scholar from database inception to 18 November 2024. The search was last updated on 10 January 2026 using the same search strategy, eligibility criteria, and screening procedures. Searches in Google Scholar were ordered by relevance, and the first 10 pages were screened for both the initial and updated searches, yielding 100 and 85 records, respectively.

The search focused on key concepts such as ‘knowledge’, ‘attitude’, ‘physiotherapist’, ‘physical therapist’, and ‘student’. Each concept was explored using indexed theme titles and free text terms. Synonyms for each concept were also searched and combined using the Boolean operator ‘OR’, while the main concepts were connected using the Boolean operator ‘AND’. Only English‐language publications were included. Full search strategies are available in Table S2.

### 2.3 Data Extraction

Database searches were conducted by one reviewer (FB). After duplicate removal, titles and abstracts were independently screened by two reviewers (FB and CM) using Covidence, followed by independent full‐text assessment by the same reviewers. FB is a physiotherapist and CM is a clinical exercise physiologist with experience in mental health, contributing domain‐specific expertise to the screening and selection process. Any discrepancies were resolved by consensus and confirmed by another investigator (SR).

One reviewer (FB) extracted all data into a predesigned Microsoft Excel data extraction form. A second reviewer (CM) independently extracted a random 30% sample to assess consistency and checked the remaining records for accuracy. Disagreements were resolved through discussion with a third author (SR).

### 2.4 Data Synthesis

Data were synthesised using a convergent segregated approach to address the research question. This method involves independently conducting quantitative and qualitative syntheses and then integrating the evidence obtained from each to achieve a more comprehensive understanding of the subject of the systematic review (Stern et al. 2020). Integration was undertaken by comparing quantitative results with qualitative themes. A joint display table was developed to map quantitative findings against qualitative themes and facilitate comparison between the two datasets (Younas et al. 2021).

Integration can result in confirmation, expansion, or discordance. Confirmation occurs when qualitative and quantitative findings converge, strengthening confidence in the results. Expansion occurs when differing findings offer complementary insights, whereas discordance arises when findings are inconsistent or conflicting (Fetters et al. 2013). Examining all three outcomes enabled integration to move beyond confirming findings towards a deeper interpretation of physiotherapists' and students' perspectives on delivering care to individuals with mental illness.

Quantitative studies and the quantitative elements of mixed‐methods research were synthesised narratively, whereas qualitative evidence was synthesised thematically. Meta‐analysis was not conducted because of substantial heterogeneity in outcome measures. Thematic synthesis followed three stages: (i) line‐by‐line coding of study findings, (ii) organisation of codes into descriptive themes, and (iii) development of analytical themes (Thomas and Harden 2008). One reviewer (FB) conducted line‐by‐line coding of the findings of the primary studies. A second reviewer (CM) reviewed a subset of the coded data to check for consistency. Any discrepancies were discussed and resolved through consensus. Descriptive themes were collaboratively developed and subsequently refined into analytical themes. These themes were then presented to the wider research team for further discussion, validation, and refinement, ensuring methodological transparency and analytical depth.

### 2.5 Quality Assessment

Due to the inclusion of diverse research types, the Mixed Methods Appraisal Tool (MMAT) was considered suitable for assessing the quality of the selected studies (Hong et al. 2018). Two reviewers (FB and CM) independently evaluated the methodological quality and risk of bias. Discrepancies in the evaluations were resolved through discussion. Studies were not excluded based on methodological quality, as appraisal was conducted to assess the quality of the available evidence rather than to determine eligibility. The outcomes of the methodological quality assessment are presented narratively within the text.

## 3 Results

**FIGURE 1:**
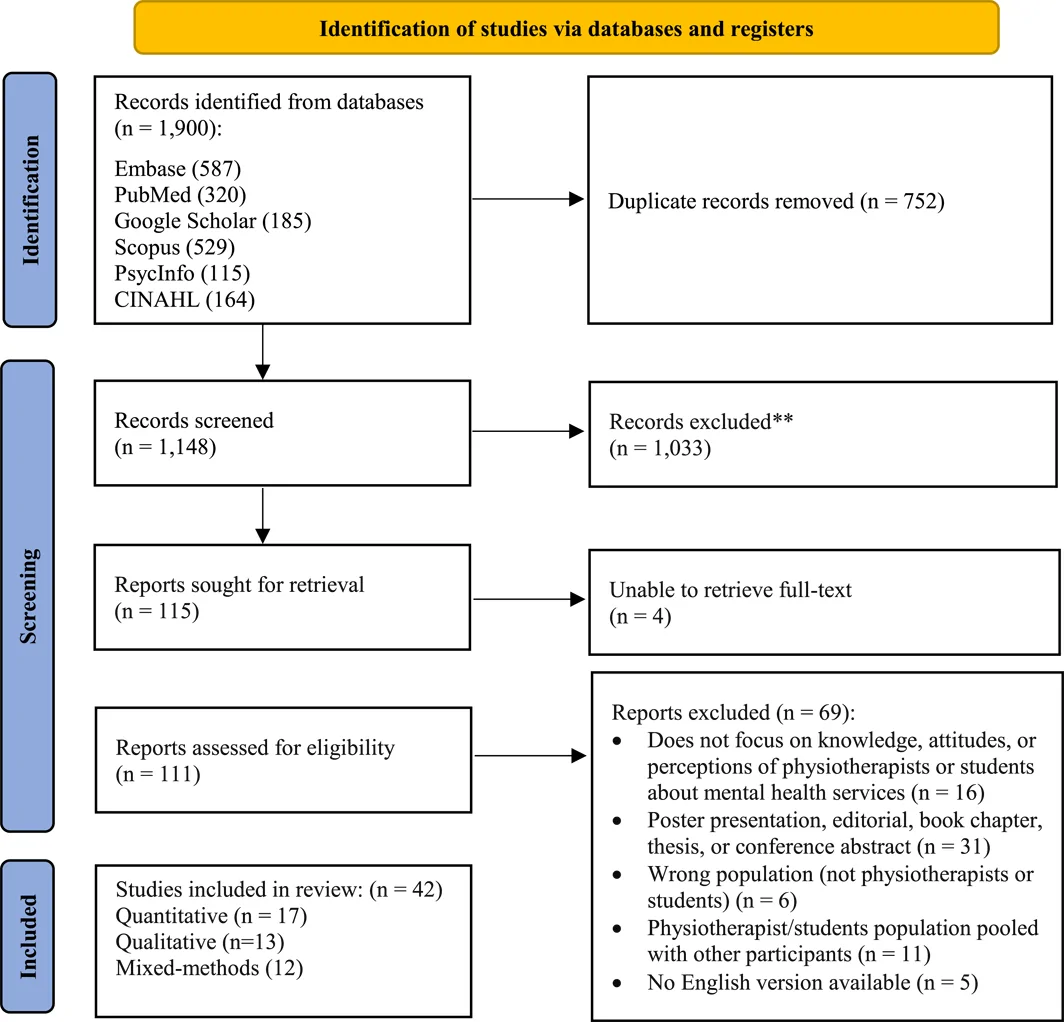
PRISMA flowchart.

An initial database search in November 2024 identified 1307 records, and an updated search in January 2026 yielded an additional 593 records. After combining both search phases in Covidence and removing 752 duplicates, 1148 records underwent title/abstract screening, resulting in the exclusion of 1033 records. Of 115 reports sought for retrieval, 111 were assessed for eligibility and 69 were excluded. Overall, 42 reports were included, including four from the January 2026 updated search. The search process is illustrated in a PRISMA flow diagram (Figure 1), with further details provided in Table S2.

### 3.1 Characteristics of the Included Studies

Of the included articles, 17 were quantitative, 13 were qualitative and 12 used mixed methods. The majority of studies were conducted in Australia (*n* = 8), followed by India (*n* = 5), South Africa (*n* = 3), Sweden (*n* = 3), Turkey (*n* = 2), the United Kingdom (*n* = 4), and the United States (*n* = 2). Additionally, one study each was conducted in Austria, Belgium, Brazil, Greece, Ireland, Italy, New Zealand, Norway, Paraguay, Saudi Arabia, Spain (*n* = 11). Four studies included physiotherapists who were members of the IOPTMH as participants.

Participants included physiotherapists (*n* = 23), students (*n* = 17), and both physiotherapists and students (*n* = 2). The mental health conditions examined encompassed a wide range of topics within the broader field of mental health and psychiatry (*n* = 30), severe and persistent mental illness (SPMI) (*n* = 1), anxiety and depression (*n* = 1), psychological distress (*n* = 3), schizophrenia (*n* = 3), eating disorders (*n* = 1), self‐harm (*n* = 1), as well as suicidal thoughts and behaviours (*n* = 2). Across all included studies, the research involved 2780 physiotherapists and 2715 students. Details of the characteristics of the included studies are presented in the Table 1.

**TABLE 1:** Characteristics of the included studies (*n* = 42).

Author (year)	Country	Design	Participants (*n*)	Key finding
Almirón et al. (2020)	Paraguay	Quantitative	Physiotherapists (*n* = 187)	Recognition of role, low confidence, and need for further training.
Alotaibi et al. (2024)	Saudi Arabia	Quantitative	Physiotherapists (*n* = 208)	Positive attitudes, limited mental health knowledge, and training needs.
Andrew et al. (2019)	Australia	Mixed‐methods	Physiotherapists (*n* = 88)	Strong role recognition and limited preparedness for managing physical conditions in people with SPMI.
Bhise et al. (2016)	India	Quantitative	Students (*n* = 94)	PT students had more positive attitudes than medical students.
Bravo et al. (2022)	Spain	Qualitative	Students (*n* = 125)	Students valued BBAT and needed more clinical practice and self‐training in the curriculum.
Byrd et al. (2025)	UK	Mixed‐methods	Students (*n* = 141)	Generally positive attitudes but low confidence.
Chiesa et al. (2024)	Italy	Quantitative	Physiotherapists (*n* = 327)	Gender influenced patient management, with female PTs showing more positive attitudes and awareness of mental health training.
Connaughton and Gibson (2016a)	Australia	Mixed‐methods	Physiotherapists (*n* = 86)	Positive attitudes towards psychiatry, with female PTs more positive than males.
Connaughton and Gibson (2016b)	Australia	Mixed‐methods	Students (*n* = 172)	Positive attitudes towards mental health, with female students more positive than males.
Dandridge et al. (2014)	UK	Quantitative	Students (*n* = 173)	Limited mental health skills and knowledge, insufficient training, and need for further education.
Danielsson et al. (2013)	Sweden	Qualitative	Physiotherapists (*n* = 10)	PTs emphasised embodied awareness to deepen patients' understanding of anxiety, supporting physiotherapy's role in mental health care.
Edgar and Connaughton (2021)	Australia	Mixed‐methods	Students (*n* = 42)	MHFA training improved attitudes towards psychiatry and confidence, with females scoring higher than males.
Furness et al. (2024)	Australia & New Zealand	Quantitative	Physiotherapists (*n* = 139)	Moderate perceived mental health knowledge and insufficient mental health content in physiotherapy curricula.
Gamblin et al. (2024)	UK	Qualitative	Students (*n* = 10)	Limited preparedness for self‐harm disclosures due to insufficient mental health education and clinical experience.
Gunduza et al. (2023)	South Africa	Quantitative	Students (*n* = 34)	Moderate knowledge, positive attitudes, and need for mental health integration in physiotherapy curricula.
Gyllensten et al. (2011)	Sweden	Quantitative	Students (*n* = 74)	Stigmatising attitudes decreased after the course, with physiotherapy students reporting less stigma towards schizophrenia than other groups.
Hooblaul et al. (2020)	South Africa	Mixed‐methods	Physiotherapists (*n* = 124)	Attitudes towards mental health were positive, with slightly higher ATP‐30 scores among females.
Hooblaul et al. (2024)	South Africa	Mixed‐method	Physiotherapists (*n* = 72) and students (*n* = 146)	Positive attitudes were reported, with higher scores among participants who treated people with mental illness daily.
Karyczak et al. (2020)	USA	Qualitative	Students (*n* = 7)	Participants reported improved attitudes towards and understanding of people with SMI following service learning.
Kazi and Ramalingam (2022)	India	Quantitative	Physiotherapists and students (*n* = 300)	The overall attitude of physiotherapists towards mental illness was positive.
Kyriakatis et al. (2025)	Greece	Quantitative	Students (*n* = 204)	Attitudes were moderately positive and associated with gender, lived experience, and close contact with someone with mental illness.
Lennon et al. (2020)	Ireland	Mixed‐methods	Physiotherapists (*n* = 320)	PTs recognised psychological distress as within their scope but felt underprepared; further mental health education increased confidence.
Lundin and Bergenheim (2020)	Sweden	Qualitative	Physiotherapists (*n* = 13)	Primary care PT frequently encounter suicidality and, despite barriers, act as therapeutic allies and potential gatekeepers for identification and referral.
McGrath et al. (2020)	Australia	Qualitative	Physiotherapists (*n* = 9)	PTs lacked confidence and role clarity after disclosures of suicidality, highlighting the need for training.
McGrath, Parnell, et al. (2024a)	Australia	Qualitative	Physiotherapists (*n* = 23)	PTs viewed psychological distress as multifactorial; mental health training is needed.
McGrath, Parnell, et al. (2024b)	Australia	Qualitative	Physiotherapists (*n* = 23)	Emotional exhaustion from frequent exposure to psychological distress and training needs.
McGrath et al. (2024)	Australia	Quantitative	Physiotherapists (*n* = 340)	Frequent encounters with psychological distress; identification was within scope, while assessment and management were viewed as beyond scope.
Moll et al. (2021)	Brazil	Qualitative	Physiotherapists (*n* = 6)	Unpreparedness to care for inpatients with mental disorders may be due to limited undergraduate psychiatry education.
Perner and Danielsson (2022)	Austria	Qualitative	Physiotherapists (*n* = 10)	Embodied learning and further training are important for developing confidence in PT mental health practice.
Prasanna et al. (2021)	India	Quantitative	Physiotherapists (*n* = 100)	Predominantly neutral attitudes, suggesting limited psychiatric exposure and training needs.
Probst and Peuskens (2010)	Belgium	Quantitative	Students (*n* = 219)	Generally positive attitudes, associated with female gender, lived experience of mental illness, and prior psychiatry education.
Rathi et al. (2023)	India	Quantitative	Students (*n* = 191)	Most students had moderately positive attitudes and good knowledge.
Singh et al. (2024)	India	Quantitative	Students (*n* = 288)	Moderate mental health knowledge and positive attitudes.
Soundy et al. (2014)	Global (IOPTMH members)	Mixed‐methods	Physiotherapists (*n* = 151)	Barriers: low patient motivation and limited professional prioritisation; facilitators: Professional support, enjoyment, and autonomy.
Soundy et al. (2016)	Global (IOPTMH members)	Mixed‐methods	Physiotherapists (*n* = 28)	Most strongly agreed that PT should play a central role in delivering physical activity to people with eating disorders.
Stubbs et al. (2014a)	Global (IOPTMH members)	Mixed‐methods	Physiotherapists (*n* = 151)	PTs identified physical activity benefits and felt equipped to lead its delivery for people with schizophrenia.
Stubbs et al. (2014b)	Global (IOPTMH members)	Mixed‐methods	Physiotherapists (*n* = 151)	PTs play a key role in schizophrenia care, with physical activity promotion central to practice.
Tehrany et al. (2024)	UK	Qualitative	Physiotherapists (*n* = 7)	Acknowledged responsibility for mental health care, but reported low confidence, limited experience, systemic barriers, and need for further training and mentorship.
Tessem et al. (2022)	Norway	Qualitative	Student (*n* = 16)	Physical activity mentoring of people with mental illness supported learning; regular, standardised supervision needed.
Yildirim and Balci (2022)	Turkey	Quantitative	Physiotherapists (*n* = 155)	More positive beliefs were associated with postgraduate education and family exposure to mental illness.
Yildirim et al. (2015)	Turkey	Quantitative	Students (*n* = 524)	Moderately positive beliefs, associated with personal contact and mental health consultation; need for further training.
Zechner et al. (2022)	USA	Qualitative	Students (*n* = 7)	Negative attitudes and unpreparedness to work with people with SMI; service learning supported student preparation.

### 3.2 Narrative Synthesis (Quantitative Studies)

#### 3.2.1 Knowledge and Confidence Related to Mental Health

Mental health knowledge varied among populations. Practising physiotherapists commonly reported knowledge gaps, limited preparation, or need for further psychiatric and mental health training (Almirón et al. 2020; Alotaibi et al. 2024; Andrew et al. 2019; Furness et al. 2024; Lennon et al. 2020). Student‐focused studies reported limited educational exposure or concerns about insufficient knowledge (Dandridge et al. 2014). In contrast, other studies found moderate to good knowledge, particularly among students with greater academic or clinical exposure (Gunduza et al. 2023; Rathi et al. 2023; Singh et al. 2024).

Confidence was examined in four studies. Limited confidence was reported in managing people with severe and persistent mental illness (Andrew et al. 2019), while additional mental health education and Mental Health First Aid (MHFA) training were associated with greater confidence (Edgar and Connaughton 2021; Lennon et al. 2020). Female physiotherapists also reported greater confidence than male physiotherapists in continuing treatment for patients with generalised anxiety disorder or previous contact with mental health professionals (Chiesa et al. 2024).

The findings indicate that knowledge and confidence are related but have distinct constructs. Confidence appeared to vary according to education, training, and participant characteristics. Differences in populations, contexts, and measurement approaches limited cross‐study comparison, and the use of adapted or custom‐developed questionnaires with limited psychometric reporting precluded data pooling.

#### 3.2.2 Attitudes and Beliefs Related to Mental Health

Twenty studies examined physiotherapists' and physiotherapy students' attitudes and beliefs regarding mental health. Attitudes towards mental illness, psychiatry, and working with people with mental health conditions were generally positive or moderately positive, although some studies reported neutral or mixed findings (Alotaibi et al. 2024; Prasanna et al. 2021). Beliefs were assessed less frequently. Physiotherapy students generally reported moderately positive beliefs towards mental illness (Yildirim et al. 2015). More favourable beliefs were associated with previous personal or professional exposure to mental health conditions (Yildirim et al. 2015; Yildirim and Balci 2022). Practising physiotherapists viewed identifying psychological distress as part of their role but were uncertain whether its assessment fell within their scope of practice (McGrath et al. 2024).

Because studies used different populations, scales, thresholds, and reporting formats, pooling of attitude scores was not undertaken. Structured teaching, psychiatry courses, mental health placements, clinical experience, and direct contact with people with mental illness were identified as factors that may improve attitudes and reduce stigma (Connaughton and Gibson 2016b; Dandridge et al. 2014; Gyllensten et al. 2011; Probst and Peuskens 2010). An overview of the outcome measures is presented in Table S3.

#### 3.2.3 Perceptions

Other studies have explored the perspectives and experiences of physiotherapists and physiotherapy students in relation to mental health practice (Soundy et al. 2014, 2016; Stubbs et al. 2014a; Stubbs et al. 2014b). Conducted with the involvement of members of the IOPTMH, these investigations offer important contributions to the understanding of physiotherapy within mental health contexts. All studies used surveys with open‐ and closed‐ended questions. Instruments were developed from the authors' expertise and assessed for face validity.

The findings from these studies revealed that participating physiotherapists typically possessed over a decade of clinical experience in mental health settings, and had received university training on schizophrenia and managing related physical comorbidities. Two studies highlighted the perceived benefits of physical activity for individuals with schizophrenia, as well as barriers and facilitators to its implementation. While physiotherapists widely recognised the importance of physical activity, gaps in practice persisted.

### 3.3 Thematic Synthesis (Qualitative Studies)

**FIGURE 2:**
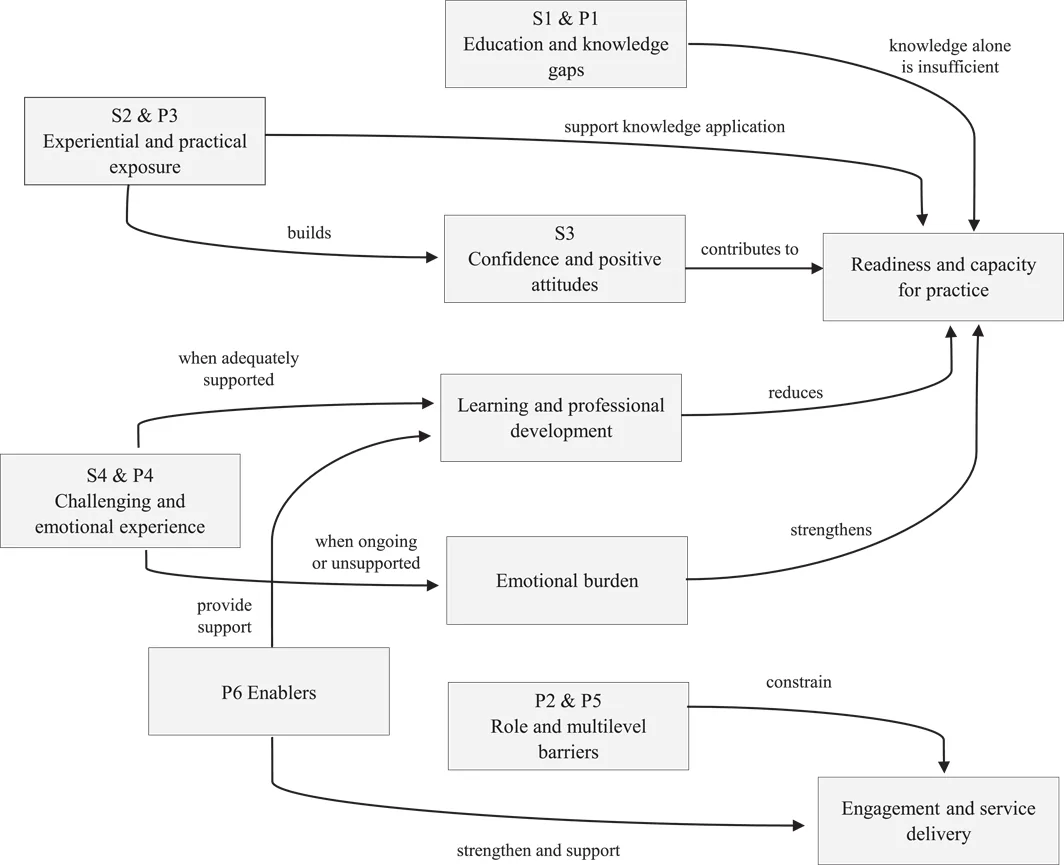
Integrated thematic model of physiotherapy practice in mental health. The figure integrates the student and practitioner themes and illustrates the conceptual relationships among knowledge, experiential learning, confidence, emotional demands, and readiness and capacity for practice. Arrows represent relationships identified through the integrated synthesis and do not imply confirmed causality. *P, practitioner; S, student.

Thematic synthesis identified a total of 10 themes, six relating to the perspectives of practicing physiotherapists and four concerning the experiences of students, as presented in Figure 2. Detailed information on the included studies and illustrative quotations for each theme are provided in Table S4.

#### 3.3.1 Practitioners' Perceptions

##### 3.3.1.1 Theme 1: Knowledge and Training Gaps

Physiotherapists often felt underprepared to address mental health issues in practice, largely due to limited exposure during their education and clinical training. Insufficient integration of mental health content in undergraduate programs contributed to uncertainty in responding to patients' psychological needs. Participants emphasised the need for foundational competencies to support more confident and competent mental health practice, including effective communication skills, recognition of common symptoms, understanding of appropriate treatments, and awareness of the relationship between physical and mental health.

There’s a distinct lack of training and our understanding of that how to treat people with severe and persistent mental illness and also our ability to communicate beyond what people have a natural affinity for doing

(Andrew et al. 2019)

##### 3.3.1.2 Theme 2: Role Perception and Professional Identity

Participants expressed varied views on physiotherapy's role in mental health care. Some were uncertain whether mental health fell within physiotherapy practice, suggesting that role ambiguity may contribute to practice gaps. Others emphasised professional boundaries, defining physiotherapists' roles as screening, support, and referral rather than counselling or specialist mental health care. Those with mental health experience reported greater confidence and a clearer professional identity.

I had no idea that I had to treat people living with mental illness after graduation; I didn’t know what my role was as a physiotherapist. I attended a conference and I heard a physiotherapist talking about mental health and I was utterly surprised

(Hooblaul et al. 2020)

##### 3.3.1.3 Theme 3: Physiotherapists' Encounters With Psychological Distress in Practice

Physiotherapists reported that patients' psychological distress was often associated with trauma, emotional difficulties, and social factors, rather than physical symptoms alone. Although only a few studies explicitly described this distress as multifactorial, the included accounts suggested that it could involve abuse, domestic violence, low mood, withdrawal from care, reduced motivation, substance use, and family‐related stress. These findings suggest that physiotherapists may need to recognise emotional and psychosocial concerns alongside physical health needs.

A lot of our patients…are coming from a history of abuse and trauma. In my practice, I have heard a lot of stories of sexual abuse and domestic violence

(McGrath et al. 2024a)

##### 3.3.1.4 Theme 4: Emotional Demands of Engaging With Mental Health Concerns

Physiotherapists described emotional burden as part of clinical engagement with patients experiencing mental health concerns, particularly when listening to disclosures of distress, trauma, or suicidal ideation. Participants reported emotional exhaustion, feeling overwhelmed, and a need to debrief after difficult encounters.

It’s one of those things where spending energy on empathizing with somebody is exhausting…You try not to take on too much of their concerns and their stress and most of the things but inevitably, it will happen when you will have a day of particularly heavy or particularly crushing, sad stories that you’ve heard

(McGrath et al. 2024b)

##### 3.3.1.5 Theme 5: Multilevel Barriers to Integrating Physiotherapy in Mental Health Care

The integration of physiotherapy into mental health care is hindered by barriers at the patient, provider, and structural levels. Patient‐level challenges include low motivation, psychological symptoms (e.g., low self‐esteem, cognitive distortions), physical limitations (e.g., medication side effects), and practical issues such as transport and financial constraints. Provider‐level barriers included limited confidence related to training and knowledge gaps in mental health and the mind‐body connection. Time constraints from structured clinical schedules were influenced by compensation models that did not account for longer consultations with patients experiencing mental health concerns. Structural barriers included limited referrals and limited multidisciplinary integration. Together, these obstacles limit the effective contribution of physiotherapy in mental health contexts.

We don’t know where we could send them and we don’t ever get asked to refer them anywhere

(Andrew et al. 2019)

##### 3.3.1.6 Theme 6: Enablers to Engaging in Physiotherapy Services

Despite the barriers to integrating physiotherapy into mental health care, several enabling factors can enhance physiotherapists' contribution. Enablers of engagement included fostering therapeutic relationships and trust, designing personalised and enjoyable interventions, and delivering multiple types of social support, such as emotional, informational, esteem‐based, and tangible support. Moreover, mentorship and clinical guidance from other professions and senior physiotherapists, along with opportunities for peer consultation, were reported as important enablers.

A good discussion around what exercise the individual is interested in and tailoring a programme around this

(Soundy et al. 2014)

#### 3.3.2 Students' Perceptions

##### 3.3.2.1 Theme 1: Perceived Gaps in Education and Training

Students expressed a sense of unpreparedness when it came to engaging with mental health in physiotherapy contexts. A common concern among students was the limited formal education and clinical exposure related to mental health. They reported insufficient training, unclear procedural guidelines, and uncertainty about the boundaries of their professional roles. Many students also noted a lack of communication skills relevant to mental health settings, pointing to the need for curriculum improvements. Furthermore, broader systemic issues, including stigma around mental illness, contributed to their lack of confidence in working in this field.

[Individuals with a serious mental illness] are definitely a population I hadn’t really thought about specifically. It’s not something that we really learn a lot about in physical therapy

(Zechner et al. 2022)

##### 3.3.2.2 Theme 2: Value of Practical and Experiential Learning

Practical and experiential learning was viewed as important for connecting theoretical knowledge with clinical practice. Learning opportunities included embodied experiences through Basic Body Awareness Therapy (BBAT), structured knowledge and communication skills training through Mental Health First Aid (MHFA), and direct practice through physical activity mentorship and service‐learning placements. Direct interaction with clients helped students develop a more integrated understanding of mental health in physiotherapy, while also increasing their confidence, comfort, empathy, and reflection in working with people with mental health problems.

If I had not experienced my supervisor pushing me to step outside of my comfort zone, I would have missed a lot of learning, I think. I learned a lot from asking difficult questions

(Tessem et al. 2022)

##### 3.3.2.3 Theme 3: Strategies to Improve Confidence and Attitudes of Students in Mental Health

Physiotherapy students often began mental health‐related practice with uncertainty, discomfort or stigmatising assumptions. Clinical exposure, supervision, and structured training were identified as important strategies to improve confidence and support more positive attitudes. Guided exposure to people with mental illness helped students feel more confident, less judgemental, and better able to understand physiotherapy's role in mental health practice.

So, I think having exposure to that (Pt with MI) would definitely increase confidence especially with guidance from a mental health physio who has a lot of knowledge in that area

(Byrd et al. 2025)

##### 3.3.2.4 Theme 4: Challenging Experiences of Working With People With Mental Illness as a Catalyst for Learning

Students described experiencing emotionally demanding and complex interactions when responding to patient behaviours as pivotal to their learning. Although emotionally taxing, these experiences fostered personal growth, empathy, and resilience. What emerged was not merely distress but a growing recognition of discomfort as a constructive force in their professional development.

Adapting your exercises based on each patient, individually tailoring things to keep up their confidence. Because sometimes you try to get your patient to do something—it might be a little too challenging and you see them kinda like knock them down or they kinda get discouraged or if they have a low frustration level

(Karyczak et al. 2020)

### 3.4 Integration of Results

Integration showed broad convergence and no clear discordance, while the qualitative findings provided complementary insights. Both syntheses identified gaps in mental health knowledge, training, and clinical exposure, while highlighting the value of education and practical experience. The qualitative findings deepened the quantitative results by showing how these gaps were experienced in practice, including under‐preparedness, role uncertainty, difficulties integrating mental health into physiotherapy care, and the emotional demands of clinical engagement. They also challenged a simple interpretation of the generally favourable attitudes reported quantitatively, indicating that positive attitudes did not necessarily translate into confidence or readiness for practice.

The combined quantitative and qualitative findings revealed two interrelated patterns. First, gaps in mental health knowledge were accompanied by uncertainty about applying that knowledge, suggesting a perceived knowledge–practice gap. Second, positive attitudes towards mental health care did not consistently align with confidence or preparedness for clinical practice. Qualitative findings provided context for these patterns, pointing to limited formal education, role ambiguity, and few opportunities to develop skills in recognising and responding to mental health concerns.

Experiential learning emerged as a potential approach to supporting students' preparedness to apply mental health knowledge and positive attitudes in practice. By providing supported opportunities to engage with complex clinical situations, this approach may facilitate the integration of conceptual understanding with psychosocially informed decision‐making. These findings provide a basis for further evaluation of experiential learning as an approach to strengthening students' perceived preparedness for mental health practice.

The qualitative findings extended the quantitative results by identifying emotional burden as a distinct dimension of workforce readiness. This dimension may not be fully captured by measures of knowledge and attitudes. Physiotherapists may therefore require preparation and support to manage the emotional demands of working with individuals with mental health conditions, including guidance on professional boundaries and access to supervision.

The qualitative themes of barriers and facilitators extended the quantitative findings by identifying broader factors influencing physiotherapists' engagement with mental health in practice. These included recognition of physiotherapists' roles by other healthcare professionals, professional support, therapeutic relationships, patient‐centred care, and wider health‐system factors. The integration of quantitative and qualitative findings is presented in the joint display in Table 2.

**TABLE 2:** Joint display of integrated quantitative and qualitative findings on physiotherapy students' knowledge, attitudes, and perceptions towards mental health.

Domain	Quantitative findings	Qualitative findings	Integrated finding	Integration type
Knowledge	Physiotherapists tended to demonstrate lower knowledge levels, while students generally showed moderate‐to‐good knowledge. Knowledge assessed using different instruments, limiting score comparability.	Both groups felt inadequately prepared to address mental health concerns, attributing this to limited formal mental health education and insufficient clinical exposure.	Moderate‐to‐good knowledge did not necessarily translate into preparedness for practice. Limited undergraduate education and clinical exposure may create a knowledge‐to‐practice gap that persists into professional practice.	Expansion
	Higher knowledge scores were associated with previous mental health experience and greater academic or clinical exposure.	Direct mental health experience helped students deepen their understanding and build confidence, empathy, and reflective skills, while supporting physiotherapists' confidence and professional identity.	Mental health experience and academic or clinical exposure were associated with higher knowledge and were perceived to support a broader understanding of mental health, greater confidence and empathy, reflective practice, and clearer professional identity.	Confirmation with expansion
Attitudes	Most studies reported favourable attitudes towards mental health, although differences in the instruments used limited direct comparison across studies.	Both students and physiotherapists described feeling underprepared or uncertain when engaging with mental health. They often perceived mental health care as beyond the physiotherapy role.	Favourable attitudes did not necessarily translate into readiness to practice, particularly where confidence and clarity about the physiotherapy role remained limited.	Expansion
	More favourable attitudes were associated with prior mental health experience and mental health education, including university courses, psychiatry training, and academic progression.	Participants described prior experience, clinical placements, and structured training as supporting confidence, communication, willingness to provide care, and engagement in mental health practice.	Experiential and practical exposure may support more favourable attitudes and perceived readiness to provide mental health care.	Confirmation with expansion
	No quantitative findings were identified on emotional demands during mental health clinical encounters.	Physiotherapists described emotional burden and a need for debriefing after difficult encounters, while students viewed emotionally demanding encounters as important learning experiences.	Emotional demands may support learning and professional development when adequately supported, but may contribute to emotional burden when exposure is ongoing or insufficiently managed.	N/A
Perceptions	One quantitative study reported barriers including lack of motivation to exercise (45%), medication side effects (27%), negative symptoms (21%), and fluctuations in mood (19%).	Patient symptoms and practical constraints increased the need for flexible care, while gaps in physiotherapists' knowledge and confidence, along with limited time, funding, referral pathways, and multidisciplinary support, reduced their capacity to respond effectively.	Patient‐level challenges increased clinical demands, while provider and system constraints limited the capacity to meet those demands. These barriers appeared to reinforce one another, potentially reducing engagement and service delivery.	Expansion
	One quantitative study reported facilitators including esteem support from healthcare professionals (28%) and patient enjoyment and autonomy (25%).	Patient‐centred care, therapeutic relationships, and social support promoted engagement, while mentorship, peer consultation, and supportive service structures strengthened physiotherapists' capacity to respond.	Enjoyment, autonomy, and professional support were consistently identified as facilitators, suggesting that engagement is strengthened when patient‐centred care is supported by therapeutic relationships and enabling service structures.	Expansion

### 3.5 Quality Assessment

Most studies presented clear research questions and appropriate designs, but some limitations were identified. In quantitative studies, issues included small or non‐representative samples, potential nonresponse bias, and limited evidence for the measurement properties of outcome measures in 13 studies. In qualitative studies, limited coherence between data sources, collection, analysis, and interpretation was identified in 5 studies. In mixed‐methods studies, adherence to the methodological quality criteria of each component was limited in 12 studies, particularly due to insufficient descriptions of analytic methods and weak integration of qualitative and quantitative findings. These limitations should be considered when interpreting this review's findings. A detailed summary of the appraisal results is provided in Table S5.

## 4 Discussion

This systematic review integrates quantitative and qualitative evidence, indicating broad recognition of the relevance of mental health to physiotherapy practice. Although attitudes were generally positive among physiotherapists and physiotherapy students, levels of mental health knowledge ranged from limited to good. Qualitative findings further indicated that some participants felt inadequately prepared to translate this knowledge into practice, particularly because of limited training, unclear professional roles, and insufficient clinical preparation.

Mental health knowledge varied and was generally higher among participants with prior education or clinical exposure. However, physiotherapists often felt underprepared to address mental health concerns and uncertain whether these fell within their scope of practice, suggesting a perceived knowledge–practice gap. Although positive attitudes provided an important foundation, they did not necessarily translate into clinical preparedness. Similar patterns have been reported in previous research, where practitioners felt underprepared to work with individuals experiencing mental health conditions (Ribeiro et al. 2024). Evidence from other mental health workforces likewise suggests that positive attitudes may co‐exist with gaps in knowledge, competence, clinical preparedness, and training (Cruciani et al. 2024).

The available evidence suggests that experiential learning may contribute to the development of mental health competence in physiotherapy education. This pattern was observed across BBAT, physical activity mentorship, and service‐learning placements, in which students' learning experiences involved participation, client interaction, and reflection. Accordingly, mental health education may benefit from complementing theoretical content with structured opportunities for practical application and direct engagement. This is consistent with previous research showing that targeted education can improve healthcare professionals' mental health literacy and behaviours (Crockett et al. 2025), while clinical placements may strengthen theory‐based learning and reduce negative attitudes and stigma (Hasan 2020).

Physiotherapists may experience emotional burden when supporting patients with mental health conditions. Compassion fatigue is the physical and emotional exhaustion arising from prolonged exposure to patients' suffering and is recognised as an occupational risk in healthcare. Although it was not directly examined in the included studies, physiotherapists working with people experiencing mental health conditions may be vulnerable to compassion fatigue and emotional exhaustion, consistent with broader healthcare literature (Marshman et al. 2022; Norrman Harling et al. 2020). Nevertheless, some student accounts suggested that emotionally challenging encounters also fostered empathy, reflection, and professional growth. This indicates a dual process in which emotional burden and learning may coexist, underscoring the need for supervision, debriefing, and boundary‐setting in mental health practice.

Several studies in this review identified patient‐, provider‐, and system‐level barriers to integrating mental health into physiotherapy practice, including time pressures, limited reimbursement, low referral rates, and insufficient multidisciplinary coordination. These findings suggest that improving physiotherapists' mental health literacy, while important, may be insufficient without practice environments that support implementation. This interpretation is consistent with research on trauma‐informed physiotherapy, where limited training, time constraints, heavy workloads, and discharge pressures were reported to constrain patient‐centred practice (S. Heywood et al. 2025). Broader organisational change and integrated care literature offers relevant considerations, including staff training, safe practice environments, collaborative decision‐making, and organisational support (Ambreen et al. 2025; Lewis et al. 2023). These implications should be interpreted cautiously as they were not directly tested in the included physiotherapy studies.

Taken together, the findings suggest an explanatory pathway in which knowledge and positive attitudes may not translate into practice without clinical exposure and opportunities to apply learning. This is consistent with Kolb's experiential learning theory, which emphasises experience, reflection, conceptualisation, and application (Kolb 2015). In addition to limited experiential learning opportunities, role ambiguity may further reduce confidence by creating uncertainty about professional boundaries and responsibilities. From a professional identity perspective, role understanding is shaped by educational and practice contexts (Rappazzo et al. 2022). Thus, experiential learning and clearer roles may strengthen confidence and support mental health practice.

Measurement limitations remain an important consideration for advancing research in this field. The included studies used varied instruments to assess knowledge and attitudes towards mental health, including established measures and study‐specific questionnaires with limited or unreported validation. Although established instruments offered some standardisation, conceptual variation and moderate internal consistency remained. Studies assessed different constructs, ranging from knowledge and attitudes to broader concepts such as confidence and beliefs, which limited comparability across outcomes. Findings on physiotherapists' and students' knowledge and attitudes should therefore be interpreted cautiously.

Drawing on diverse study designs, this review provides a comprehensive synthesis of evidence on physiotherapy and mental health. However, some limitations should be noted. The included studies varied in design, outcome measures, conceptual definitions, and methodological quality. This variation may reduce comparability and generalisability. In particular, limitations related to sampling, response bias, measurement quality, and integration in mixed‐methods studies may affect confidence in interpreting the findings. Although the mixed‐methods approach strengthened interpretive depth, the synthesis may still be subject to interpretive subjectivity. The predominance of English‐language sources may also limit its applicability to non‐English‐speaking contexts. Additionally, as Google Scholar was used as a supplementary source and screening was limited to the first 10 pages of results, some relevant studies may not have been identified.

Building on the gaps identified in this review, future research should prioritise the validation and, where warranted, development of physiotherapy‐specific measures relevant to mental health practice. Longitudinal and intervention studies are needed to evaluate whether and how educational models incorporating experiential learning facilitate the translation of knowledge into clinical practice. Implementation research should further investigate how service structures and multidisciplinary collaboration influence physiotherapists' engagement in mental health care.

### 4.1 Implications of Physiotherapy Practice

This review identifies gaps in physiotherapists' and physiotherapy students' knowledge, role clarity, and preparedness for mental health care. Although attitudes were generally positive, many participants reported feeling insufficiently equipped to apply mental health knowledge in practice. These findings suggest that physiotherapy education should prioritise training in communication, recognition of psychological distress, role boundaries, and referral. Curricula may consider incorporating experiential learning through supervised clinical placements, simulations, and case‐based discussions to support students' preparedness for mental health care. Similar practice‐based continuing professional development may further strengthen clinicians' confidence and preparedness to address mental health needs.

Limited understanding of physiotherapists' role in mental health care was identified as a potential barrier to interdisciplinary collaboration. The findings support the consideration of clearer role definitions and interprofessional education to facilitate collaboration and appropriate referral. Physiotherapists may also benefit from support in managing the emotional demands of working with patients experiencing mental health challenges. This support could include clinical supervision, structured debriefing, peer support, and clear referral pathways.

Importantly, structural barriers, including time pressures, reimbursement models, and limited multidisciplinary coordination, suggest that educational initiatives should be accompanied by broader practice‐ and system‐level changes. Alongside these changes, promoting the view that ‘mental health is every physiotherapist's business’ could foster a shift in professional culture and support the integration of psychosocial approaches into routine practice.

## Author Contributions

The study was conceptualised and designed by F.B., S.R., D.V., H.S., G.M., and D.P. F.B. undertook the literature search, while both F.B. and C.M. were responsible for screening, data analysis, and quality assessment. The initial draft of the manuscript was prepared by F.B. and S.R. All authors: F.B., C.M., G.M., D.V., H.S., D.P., A.A., H.F., B.S. and S.R. contributed to the critical review and editing of the manuscript. Study supervision: S.R., D.V., G.M., H.S. and D.P. All authors have read and approved the final version of the manuscript.

## Funding

The author, FB, was funded by Lembaga Pengelola Dana Pendidikan (Indonesia Endowment Fund for Education), Ministry of Finance of the Republic of Indonesia, Award Number: 202401220800156.

## Ethics Statement

The authors have nothing to report.

## Conflicts of Interest

The authors declare no conflicts of interest.

## Supporting information


**Tabel S1:** PRISMA 2020 checklist.

**Table S2:** Search Strategy and Final Integrated Study‐Selection Process.

**Table S3:** Psychometric Properties of Measures Assessing Knowledge, Attitudes, and Perceptions.

**Table S4:** Illustrative quotations and references reporting each theme.

**Table S5:** Assessment of the Methodological Quality of the Included Studies Using the MMAT.

## Data Availability

All relevant materials, including search strategies, psychometric properties, and quality assessment, are provided in the Supplementary Materials.
