# Supplementary material for: Physiotherapy in Mental Health: A Mixed Methods Systematic Review of Practitioner and Student Knowledge, Attitudes, and Perceptions
Source: Physiother Res Int. 2026 Aug 24;31(4):e70329. doi: 10.1002/pri.70329 (PMC13501346; doi:10.1002/pri.70329)
Supplement: Supplementary file 1 [file PRI-31-e70329-s001.docx]

**Table S2. Search Strategy and Final Integrated Study-Selection Process**

**Keywords for databases**

Initial Search (November 2024)

| No | Database | Keywords | Articles | Access Date |
| --- | --- | --- | --- | --- |
| 1 | EMBASE - OVID | (physiotherap*.tw. OR "physical therapy".tw. OR "physical therap*".tw. OR (student*.tw. AND (physiotherap*.tw. OR "physical therapy".tw. OR "physical therap*".tw.))) AND (knowledge.tw. OR belief*.tw. OR attitude*.tw. OR perception*.tw. OR view*.tw.) AND ("mental health".tw. OR "mental illness".tw. OR psychiatry.tw.) | 429 | 18/11/2024 |
| 2 | PubMed | (physiotherap*[tiab] OR "physical therapy"[tiab] OR "physical therap*"[tiab] OR (student*[tiab] AND (physiotherap*[tiab] OR "physical therapy"[tiab] OR "physical therap*"[tiab]))) AND (knowledge[tiab] OR belief*[tiab] OR attitude*[tiab] OR perception*[tiab] OR view*[tiab]) AND ("mental health"[tiab] OR "mental illness"[tiab] OR psychiatry[tiab]) | 243 | 18/11/2024 |
| 3 | Scopus | (TITLE-ABS(physiotherap*) OR TITLE-ABS("physical therapy") OR TITLE-ABS("physical therap*") OR (TITLE-ABS(student*) AND (TITLE-ABS(physiotherap*) OR TITLE-ABS("physical therapy") OR TITLE-ABS("physical therap*")))) AND (TITLE-ABS(knowledge) OR TITLE-ABS(belief*) OR TITLE-ABS(attitude*) OR TITLE-ABS(perception*) OR TITLE-ABS(view*)) AND (TITLE-ABS("mental health") OR TITLE-ABS("mental illness") OR TITLE-ABS(psychiatry)) | 286 | 18/11/2024 |
| 4 | PsycInfo | (physiotherap*.ti,ab. OR "physical therapy".ti,ab. OR "physical therap*".ti,ab. OR (student*.ti,ab. AND (physiotherap*.ti,ab. OR "physical therapy".ti,ab. OR "physical therap*".ti,ab.))) AND (knowledge.ti,ab. OR belief*.ti,ab. OR attitude*.ti,ab. OR perception*.ti,ab. OR view*.ti,ab.) AND ("mental health".ti,ab. OR "mental illness".ti,ab. OR psychiatry.ti,ab.) | 102 | 18/11/2024 |
| 5 | CINAHL | ((TI physiotherap* OR AB physiotherap*) OR (TI "physical therapy" OR AB "physical therapy") OR (TI "physical therap*" OR AB "physical therap*") OR ((TI student* OR AB student*) AND ((TI physiotherap* OR AB physiotherap*) OR (TI "physical therapy" OR AB "physical therapy") OR (TI "physical therap*" OR AB "physical therap*")))) AND ((TI knowledge OR AB knowledge) OR (TI belief* OR AB belief*) OR (TI attitude* OR AB attitude*) OR (TI perception* OR AB perception*) OR (TI view* OR AB view*)) AND ((TI "mental health" OR AB "mental health") OR (TI "mental illness" OR AB "mental illness") OR (TI psychiatry OR AB psychiatry)) | 147 | 18/11/2024 |
| 6 | Google Scholar | physiotherapy OR "physical therapy” OR "physiotherapy students" AND knowledge OR belief OR attitude OR perception OR view AND “mental health" OR "mental illness" OR psychiatry | 100 | 18/11/2024 |
|  | Total |  | 1,307 |  |

Updated 2026 (January 2026)

| No | Database | Keywords | Articles | Access Date |
| --- | --- | --- | --- | --- |
| 1 | EMBASE - OVID | (physiotherap*.tw. OR "physical therapy".tw. OR "physical therap*".tw. OR (student*.tw. AND (physiotherap*.tw. OR "physical therapy".tw. OR "physical therap*".tw.))) AND (knowledge.tw. OR belief*.tw. OR attitude*.tw. OR perception*.tw. OR view*.tw.) AND ("mental health".tw. OR "mental illness".tw. OR psychiatry.tw.) | 158 | 10/01/2026 |
| 2 | PubMed | (physiotherap*[tiab] OR "physical therapy"[tiab] OR "physical therap*"[tiab] OR (student*[tiab] AND (physiotherap*[tiab] OR "physical therapy"[tiab] OR "physical therap*"[tiab]))) AND (knowledge[tiab] OR belief*[tiab] OR attitude*[tiab] OR perception*[tiab] OR view*[tiab]) AND ("mental health"[tiab] OR "mental illness"[tiab] OR psychiatry[tiab]) | 77 | 10/01/2026 |
| 3 | Scopus | (TITLE-ABS(physiotherap*) OR TITLE-ABS("physical therapy") OR TITLE-ABS("physical therap*") OR (TITLE-ABS(student*) AND (TITLE-ABS(physiotherap*) OR TITLE-ABS("physical therapy") OR TITLE-ABS("physical therap*")))) AND (TITLE-ABS(knowledge) OR TITLE-ABS(belief*) OR TITLE-ABS(attitude*) OR TITLE-ABS(perception*) OR TITLE-ABS(view*)) AND (TITLE-ABS("mental health") OR TITLE-ABS("mental illness") OR TITLE-ABS(psychiatry)) | 243 | 10/01/2026 |
| 4 | PsycInfo | (physiotherap*.ti,ab. OR "physical therapy".ti,ab. OR "physical therap*".ti,ab. OR (student*.ti,ab. AND (physiotherap*.ti,ab. OR "physical therapy".ti,ab. OR "physical therap*".ti,ab.))) AND (knowledge.ti,ab. OR belief*.ti,ab. OR attitude*.ti,ab. OR perception*.ti,ab. OR view*.ti,ab.) AND ("mental health".ti,ab. OR "mental illness".ti,ab. OR psychiatry.ti,ab.) | 13 | 10/01/2026 |
| 5 | CINAHL | ((TI physiotherap* OR AB physiotherap*) OR (TI "physical therapy" OR AB "physical therapy") OR (TI "physical therap*" OR AB "physical therap*") OR ((TI student* OR AB student*) AND ((TI physiotherap* OR AB physiotherap*) OR (TI "physical therapy" OR AB "physical therapy") OR (TI "physical therap*" OR AB "physical therap*")))) AND ((TI knowledge OR AB knowledge) OR (TI belief* OR AB belief*) OR (TI attitude* OR AB attitude*) OR (TI perception* OR AB perception*) OR (TI view* OR AB view*)) AND ((TI "mental health" OR AB "mental health") OR (TI "mental illness" OR AB "mental illness") OR (TI psychiatry OR AB psychiatry)) | 17 | 10/01/2026 |
| 6 | Google Scholar | physiotherapy OR "physical therapy” OR "physiotherapy students" AND knowledge OR belief OR attitude OR perception OR view AND “mental health" OR "mental illness" OR psychiatry | 85 | 10/01/2026 |
|  | Total |  | 593 |  |

**Integrated Study-Selection Process**

| Search phase | Search date | Records identified | Included reports |
| --- | --- | --- | --- |
| Initial search | November 2024 | 1,307 | 38 |
| Updated search | January 2026 | 593 | 4 |
| Final integrated review | November 2024-January 2026 | 1,900 | **42** |

**Note**: *Records retrieved in November 2024 and January 2026 were imported into Covidence and de-duplicated across the complete dataset.*
