# Supplementary material for: Physiotherapy in Mental Health: A Mixed Methods Systematic Review of Practitioner and Student Knowledge, Attitudes, and Perceptions
Source: Physiother Res Int. 2026 Aug 24;31(4):e70329. doi: 10.1002/pri.70329 (PMC13501346; doi:10.1002/pri.70329)
Supplement: Supplementary file 2 [file PRI-31-e70329-s002.docx]

**Table S5. Assessment of the Methodological Quality of the Included Studies Using the MMAT**

| No | Quantitative descriptive | Are there clear research questions? | Do the collected data allow to address the research questions? | Is the sampling strategy relevant to address the research question? | Is the sample representative of the target population? | Are the measurements appropriate? | Is the risk of nonresponse bias low? | Is the statistical analysis appropriate to answer the research question? | Comments |
| --- | --- | --- | --- | --- | --- | --- | --- | --- | --- |
| 1 | Alotaibi et al. (2024) | Yes | Yes | Yes | No | Cant' tell | No | Yes | convenience sample, two-thirds of the calculated sample was obtained, Cronbach’s alpha test not reported |
| 2 | Bhise et al. (2016) | Yes | Yes | No | No | Yes | No | Yes | small sample size, data from one university only |
| 3 | Chiesa et al. (2024) | Yes | Yes | Yes | No | No | No | Yes | low response rate (30%), non-validated questionnaire |
| 4 | Dandridge et al. (2014) | Yes | Yes | Yes | No | Yes | No | Yes | very low response rate, questionnaire developed for study and only face validity available, inclusion/exclusion criteria limited |
| 5 | Furness et al. (2024) | Yes | Yes | Yes | No | No | Can't tell | Yes | questionnaire developed for study and only face validity available, response rate not reported |
| 6 | Gunduza et al. (2023) | Yes | Yes | No | No | Yes | No | Yes | purposive sampling, very low response rate (34%) |
| 7 | Kazi & Ramalingam (2022) | Yes | Yes | No | No | Can't tell | No | Yes | psychometric properties were not reported, details on sample measures were not provided, and response rate was not provided |
| 8 | Kyriakatis et al. (2025) | Yes | Yes | No | No | Yes | Can't tell | Yes | response rate was not available because the survey was distributed widely to an unknown population number |
| 9 | McGrath, Verdon, et al. (2024) | Yes | Yes | Yes | Yes | Yes | Yes | Yes | - |
| 10 | Prasanna et al. (2021) | Yes | Yes | No | No | Yes | No | Yes | convenient sampling method, population not clear, response rate not reported |
| 11 | Rathi et al. (2023) | Yes | Yes | No | No | Yes | No | Yes | only one university sampled, response rate was 62.62% |
| 12 | Singh et al. (2024) | Yes | Yes | No | No | Yes | No | Can't tell | inclusion/exclusion criteria limited, 100% response rate reported however states, "only students who were eager to participate received forms |
| 13 | Yildirim & Balci (2022) | Yes | Yes | No | No | Yes | No | Yes | acknowledged inability to calculate response rate, inclusion/exclusion criteria limited |
| 14 | Yildirim et al. (2015) | Yes | Yes | No | No | Yes | No | Yes | inclusion/exclusion criteria limited, 65% response rate, sample from two universities |

| No | Quantitative Randomised | Are there clear research questions? | Do the collected data allow to address the research questions? | Is randomization appropriately performed? | Are the groups comparable at baseline? | Are there complete outcome data? | Are outcome assessors blinded to the intervention provided? | Did the participants adhere to the assigned intervention? | Comments |
| --- | --- | --- | --- | --- | --- | --- | --- | --- | --- |
| 15 | Probst & Peuskens (2010) | Yes | Yes | Can't tell | Can't tell | No | Can't tell | No | 67% analysis (intervention group) |

| No | Quantitative Non-Randomised | Are there clear research questions? | Do the collected data allow to address the research questions? | Are the participants representative of the target population? | Are measurements appropriate regarding both the outcome and intervention (or exposure)? | Are there complete outcome data? | Are the confounders accounted for in the design and analysis? | During the study period, is the intervention administered (or exposure occurred) as intended? | Comments |
| --- | --- | --- | --- | --- | --- | --- | --- | --- | --- |
| 16 | Gyllensten et al. (2011) | Yes | Yes | Can't tell | Yes | Yes | Can't tell | Yes | total population was not reported, sampling procedures were not described |

| No | Mixed-method | Are there clear research questions? | Do the collected data allow to address the research questions? | Is there an adequate rationale for using a mixed methods design to address the research question? | Are the different components of the study effectively integrated to answer the research question? | Are the outputs of the integration of qualitative and quantitative components adequately interpreted? | Are divergences and inconsistencies between quantitative and qualitative results adequately addressed? | Do the different components of the study adhere to the quality criteria of each tradition of the methods involved? | Comments |
| --- | --- | --- | --- | --- | --- | --- | --- | --- | --- |
| 17 | Almirón et al. (2020) | Yes | Yes | Can't tell | Can't tell | Yes | Can't tell | No | inclusion/exclusion criteria limited, statistical analysis methodology description limited, response rate not reported |
| 18 | Andrew et al. (2019) | Yes | Yes | Yes | Yes | Yes | Yes | No | response rate not reported |
| 19 | Byrd et al. (2025) | Yes | Yes | Yes | Yes | Yes | Yes | No | Low response rate (estimated 9.4%), part of the survey lack validation |
| 20 | Connaughton & Gibson (2016a) | Yes | Can't tell | Can't tell | Can't tell | Yes | Yes | No | limited sample size, participant bias, qualitative analysis method lacked detail and results somewhat lacked structure and thus clarity, inclusion/exclusion criteria limited, further integration of mixed methods data would have benefited the study |
| 21 | Connaughton & Gibson (2016b) | Yes | Yes | Can't tell | Yes | Yes | Can't tell | No | limited sample, qualitative analysis method and results lacked detail, further integration of mixed methods data would have benefited the study |
| 22 | Edgar & Connaughton (2021) | Yes | Yes | Can't tell | Yes | Yes | Can't tell | No | limited sample size, no control group, several confounders |
| 23 | Hooblaul et al. (2020) | Yes | Yes | No | Yes | Yes | Can't tell | No | inclusion/exclusion criteria limited, response rate=81%, |
| 24 | Hooblaul et al. (2024) | Yes | Yes | No | Yes | Yes | Yes | Yes | inclusion/exclusion criteria limited, minimal integration of qualitative and quantitative components |
| 25 | Lennon et al. (2020) | Yes | Yes | Can't tell | Can't tell | Yes | Can't tell | No | very low response rate (10%), non-validated questionnaire, the final mixed-methods criterion was rated lower due to the quantitative component quality |
| 26 | Soundy et al. (2014) | Yes | Yes | Can't tell | Yes | Yes | Can't tell | No | questionnaire developed for study and only face validity available, response rate varied with some items having adequate response rates (60%+) and others very low (5-10%) |
| 27 | Soundy et al. (2016) | Yes | Yes | Can't tell | Yes | Yes | Can't tell | No | questionnaire developed for study and only face validity available |
| 28 | Stubbs, Soundy, Probst, De Hert, De Herdt, Parker, et al. (2014) | Yes | Yes | Can't tell | Yes | Yes | Can't tell | No | questionnaire developed for study and only face validity available, inclusion/exclusion criteria limited, response rate not reported |
| 29 | Stubbs, Soundy, Probst, De Hert, De Herdt, & Vancampfort (2014) | Yes | Yes | Can't tell | Yes | Yes | Can't tell | No | non-validated questionnaire, acknowledged, "it is not possible to determine the exact response rate and it is likely that it is relatively low and it is likely that there was a high social desirability bias in respondents." |

| No | Qualitative | Are there clear research questions? | Do the collected data allow to address the research questions? | Is the qualitative approach appropriate to answer the research question? | Are the qualitative data collection methods adequate to address the research question? | Are the findings adequately derived from the data? | Is the interpretation of results sufficiently substantiated by data? | Is there coherence between qualitative data sources, collection, analysis and interpretation? | Comments |
| --- | --- | --- | --- | --- | --- | --- | --- | --- | --- |
| 30 | Bravo et al. (2022) | Yes | Yes | Yes | Yes | Yes | Can't tell | Can't tell | study would have benefited by linking quotes to interpretation further rather than presenting each mostly |
| 31 | Danielsson et al. (2013) | Yes | Yes | Yes | Yes | Yes | Yes | Yes | - |
| 32 | Gamblin et al. (2024) | Yes | Yes | Yes | Yes | Yes | Yes | Yes | - |
| 33 | Karyczak et al. (2020) | Yes | Yes | Yes | Yes | Yes | Yes | Can’t tell | results limited with most findings presented in a table with limited explanation in-text |
| 34 | Lundin & Bergenheim (2020) | Yes | Yes | Yes | Yes | Yes | Yes | Yes | - |
| 35 | McGrath et al. (2020) | Yes | Yes | Yes | Yes | Yes | Yes | Can’t tell | small sample size |
| 36 | McGrath, Parnell, et al. (2024a) | Yes | Yes | Yes | Yes | Yes | Yes | Yes | - |
| 37 | McGrath, Parnell, et al. (2024b) | Yes | Yes | Yes | Yes | Yes | Yes | Yes | - |
| 38 | Moll et al. (2021) | Yes | Yes | Yes | Yes | Yes | Yes | Can't tell | - |
| 39 | Perner & Danielsson, (2022) | Yes | Yes | Yes | Yes | Yes | Yes | Yes | small sample size |
| 40 | Tehrany et al. (2024) | Yes | Yes | Yes | Yes | Yes | Yes | Yes | small sample size |
| 41 | Tessem et al. (2022) | Yes | Yes | Yes | Yes | Yes | Yes | Yes | - |
| 42 | Zechner et al. (2022) | Yes | Yes | Yes | Yes | Yes | Yes | Can’t tell | results limited with most findings presented in a table with limited explanation in-text |
