# Supplementary material for: Physiotherapy in Mental Health: A Mixed Methods Systematic Review of Practitioner and Student Knowledge, Attitudes, and Perceptions
Source: Physiother Res Int. 2026 Aug 24;31(4):e70329. doi: 10.1002/pri.70329 (PMC13501346; doi:10.1002/pri.70329)
Supplement: Supplementary file 4 [file PRI-31-e70329-s004.docx]

**Table S3. Psychometric Properties of Measures Assessing Knowledge, Attitudes, and Perceptions**

| Item | Authors (year) | Participant | Outcome measures | Reliability/Validity |
| --- | --- | --- | --- | --- |
| Knowledge | Almirón et al. (2020) | Physiotherapist | Adapted items from previous research (Andrew et al., 2019 - Spanish version) | Not reported (the translation and adaptation of the instrument was carried out with the authorisation of the original authors). |
|  | Alotaibi et al. (2024) | Physiotherapist | Adapted questions from previously published research (Andrew et al., 2019) | The survey was validated by experts for content relevance and by PNU physiotherapy students for clarity. Its reliability was confirmed using Cronbach's alpha test. |
|  | Andrew et al. (2019) | Physiotherapist | Custom-developed questionnaire (web-based survey) | Not reported |
|  | Chiesa et al. (2024) | Physiotherapist | Custom-developed questionnaire (ad hoc questionnaire) | Not reported |
|  | Dandridge et al. (2014) | Student | Custom-developed questionnaire | Not reported |
|  | Edgar & Connaughton (2021) | Student | Custom-developed questionnaire | Not reported |
|  | Furness et al. (2024) | Physiotherapist | Custom-developed questionnaire | Face Validity |
|  | Gunduza et al. (2023) | Student | Mental Health Knowledge Schedule (MAKS) | The test-retest reliability of 0.71 Lin’s concordance statistic and an overall internal consistency of 0.65 (Cronbach’s α). |
|  | Lennon et al. (2020) | Physiotherapist | Custom-developed questionnaire | Not reported |
|  | Rathi et al. (2023) | Student | Pre-validated questionnaires | Not reported |
|  | Singh et al. (2024) | Student | Mental Health Knowledge Schedule (MAKS) | Moderate reliability (Cronbach's alpha = 0.56), mediocre sampling adequacy (KMO = 0.632), and explained 31.58% of the variance. |
| Attitudes | Alotaibi et al. (2024) | Physiotherapist | Adapted items from previous research (Andrew et al., 2019) | The survey’s content validity was confirmed by experts for cultural relevance, and face validity was checked by physiotherapy students at PNU for clarity. Reliability was evaluated using Cronbach’s alpha. |
|  | Bhise et al. (2016) | Student | ATP-30 questionnaire | High face, concurrent, and construct validity with split-half reliability. |
|  | Byrd et al., (2025) | Student | MICA-4 (Mental Illness Clinicians’ Attitude Scale) | Previously validated (Gabbidon et al., 2013). |
|  | Chiesa et al. (2024) | Physiotherapist | Custom-developed questionnaire | Not reported |
|  | Connaughton & Gibson (2016a) | Physiotherapist | ATP-30 questionnaire | The tool has been shown to be both reliable and valid. |
|  | Connaughton & Gibson (2016b) | Student | ATP-30 questionnaire | The tool has been shown to be both reliable and valid. |
|  | Dandridge et al. (2014) | Student | Custom-developed questionnaire | Not reported |
|  | Gunduza et al. (2023) | Student | MICA-4 (Mental Illness Clinicians’ Attitude Scale) | The test–retest reliability is high at 0.80, internal consistency is 0.79 (Cronbach’s α) with a split half correlation of 0.73. |
|  | Gyllensten et al. (2011) | Student | Fear and Behavioural Intentions Toward the Mentally Ill (FABI) and the Changing Minds: Every Family in the Land questionnaire | A Swedish version, developed with the authors’ permission, showed acceptable test-retest reliability and internal consistency. |
|  | Hooblaul et al. (2020) | Physiotherapist | ATP-30 questionnaire | The tool has been shown to be reliable and valid with medical and occupational therapy students (Burra et al. 1982). |
|  | Hooblaul et al. (2024) | Student and Physiotherapist | ATP-30 questionnaire | Demonstrating reliability and validity (Burra et al. 1982; Probst & Peuskens 2010). |
|  | Kazi & Ramalingam (2022) | Student and Physiotherapist | Attitude Scale for Mental Illness (ASMI) | Not reported |
|  | Kyriakatis et al. (2025) | Student | ATP-30 questionnaire | The scale has established reliability and validity (Burra et al., 1982; Kuhnigk et al., 2007) |
|  | Prasanna et al. (2021) | Physiotherapist | MICA-4 (Mental Illness Clinicians’ Attitude Scale) | Internal consistency: Cronbach’s ⍶ with an acceptable value of ⍶ > 0.7 but not exceeding 0.9 (indicative of good internal consistency). |
|  | Probst & Peuskens (2010) | Student | ATP-30 questionnaire | Good reliability (Cronbach’s α = 0.85; split-half r = 0.84, p < 0.01; half-to-half r = 0.73, p < 0.01). |
|  | Rathi et al. (2023) | Student | MICA-4 (Mental Illness Clinicians’ Attitude Scale) | Not reported |
|  | Singh et al. (2024) | Student | MICA-4 (Mental Illness Clinicians’ Attitude Scale) | Good reliability (Cronbach's alpha = 0.72–0.79) and strong test-retest reliability (0.80). |
|  | McGrath, Verdon, et al. (2024) | Physiotherapist | Modified Physician Belief Scale (mPBS) | Acceptable reliability (α = 0.75 and 0.79), and the full scale demonstrated good reliability (α = 0.85). |
|  | Yildirim & Balci (2022) | Physiotherapist | Beliefs Toward Mental Illness (BMI) scale | Original version: Reliability was high (α = 0.89–0.91; Hirai & Clum, 2000). The Turkish version showed good internal consistency (α = 0.82; subscales: 0.71–0.80). |
|  | Yildirim et al. (2015) | Student | Beliefs Toward Mental Illness (BMI) scale | Original version: Cronbach's alpha of 0.89 for American students and 0.91 for Asian students (high internal consistency). |
| Perceptions | Soundy et al. (2016) | Physiotherapist | Custom-developed questionnaire | Face validity |
|  | Soundy et al. (2014) | Physiotherapist | Custom-developed questionnaire | Face validity |
|  | Stubbs, Soundy, Probst, De Hert, De Herdt, Parker, et al. (2014) | Physiotherapist | Custom-developed questionnaire (Vancampfort et al., 2010; Vancampfort  et al., 2012) | Face Validity |
|  | Stubbs, Soundy, Probst, De Hert, De Herdt, & Vancampfort (2014) | Physiotherapist | Custom-developed questionnaire (Stubbs, Soundy, Probst, De Hert, De Herdt, & Vancampfort, 2014) | Face Validity |
