# Supplementary material for: Physiotherapy in Mental Health: A Mixed Methods Systematic Review of Practitioner and Student Knowledge, Attitudes, and Perceptions
Source: Physiother Res Int. 2026 Aug 24;31(4):e70329. doi: 10.1002/pri.70329 (PMC13501346; doi:10.1002/pri.70329)
Supplement: Supplementary file 5 [file PRI-31-e70329-s005.docx]

**Table S4**

**Illustrative Quotations and References Reporting Each Theme**

**Physiotherapist**

| Themes | Participants quotations from primary studies | Reference |
| --- | --- | --- |
| Theme 1: Knowledge and Training Gaps | “There’s a distinct lack of training and our understanding of that [how to treat people with SPMI] and also our ability to communicate beyond what people have a natural affinity for doing” | Andrew et al. (2019)  Physiotherapists' views about providing physiotherapy services to people with severe and persistent mental illness: a mixed methods study |
|  | “lack of knowledge as to what contributions PT can make” | Soundy et al. (2014)  Barriers to and facilitators of physical activity among persons with schizophrenia: a survey of physical therapists |
|  | “I don’t believe we have knowledge in that. Just because we have time and are interested, good citizens…well, many times you are told that “That is enough!” just to listen and be present. But that is not what we are educated to do!” | Lundin & Bergenheim (2020) Encountering suicide in primary healthcare rehabilitation: the experiences of physiotherapists |
|  | “It's very hard to get confidence in the fact that it is actually your job, and you do actually have a role to play in assessing people's mental health. Second of all, you don't have any confidence in what to actually do and how to actually do it. . . As for my education, there was zero mention of [suicidal thoughts and behaviours]. The closest to that sort of thing when I was at uni was you should do your Mental Health First Aid.” | McGrath et al. (2020)  Trust, conversations and the ‘middle space’: A qualitative exploration of the experiences of physiotherapists with clients with suicidal thoughts and behaviours |
|  | “We need more education on the importance of psychological distress and how to address it. It is not sufficient to say it is beyond our scope of practice when so many patients with persistent pain display psychological distress. We cannot separate mind from body” | Lennon et al. (2020)  Psychological distress among patients attending physiotherapy: A survey-based investigation of Irish physiotherapists’ current practice and opinions. |
|  | “No exposure at the undergraduate level will lead to negative perceptions and fear of PLWMI. Mental illness has always been there, but we shy away from conditions we don’t understand”  “Not sure how I can treat a patient just with a mental illness” | Hooblaul et al. (2020)  The knowledge, attitudes and perceptions of physiotherapists in KwaZulu-Natal, South Africa, towards mental health. |
|  | “How poorly prepared we are as undergraduate physiotherapists to work with patients with psychiatric issues. Although you might not specialize in this area, you will treat patients with psychiatric conditions; therefore, some basic skills are required” | Connaughton & Gibson (2016)  Do physiotherapists have the skill to engage in the “psychological” in the bio-psychosocial approach? |
|  | “I had psychology, but I don’t think it addressed mental health. I really don’t remember now”  “This! Sometimes, not, because there are some mild disorders, sometimes the guy is depressed, we can manage it; if he’s aggressive, then we’re not so prepared. In fact, there is no preparation”  “I don’t feel prepared to care for this patient” | Moll et al (2021)  Physiotherapists and Psychiatric Hospitalization in General Hospitals: Care and Professional Training |
|  | “I think there probably is a lack of confidence and there’s a bit again, it’s a fear that people are worried that if they ask questions about mental health and that kind of opened up a can of worms and they won’t be qualified and they don’t know what to do and how to deal with it.”  “ I guess it [training] would have to be something which is applicable to us. … I think that’ll be really good to have an overview of all of those [MH conditions], and those that are that are managed medically… the medications which they are on, which they take, can that affect them in a physical way?” | Tehrany et al. (2024)  Allied health professionals’ experiences and views towards improving musculoskeletal services in the UK for patients with musculoskeletal and co-existing mental health conditions: a qualitative study. |
|  | “I cannot meet the patient in a neutral way if I´m not aware of myself. That´s the main issue. It´s really challenging to take a look at oneself. It is challenging and a long process, it´s not like: ‘well okay, I just learn and then do this job’. It is actually the personal development of a certain way of being (Wesensbildung)”. | Perner, S. and Danielsson, L., 2022. Exploring clinical reasoning in Austrian mental health physiotherapy: the physiotherapist´ s perspective. |
| Theme 2: Role perception and professional identity | “I had no idea that I had to treat PLWMI after graduation; I didn’t know what my role was as a physiotherapist. I attended a conference and I heard a physiotherapist talking about mental health and I was utterly surprised” | Hooblaul et al. (2020)  The knowledge, attitudes and perceptions of physiotherapists in KwaZulu-Natal, South Africa, towards mental health |
|  | “We sometimes do get caught up in becoming counsellors, and we're not equipped to be counsellors or mental health workers. We're good at, I think, screening and being able to see if somebody's at risk [of suicide] …. Hopefully [we are also good at referring] them [to a mental health professional], but knowing our limitations is so important” | McGrath et al. (2020)  Trust, conversations and the ‘middle space’: A qualitative exploration of the experiences of physiotherapists with clients with suicidal thoughts and behaviours |
|  | “don’t understand a lot about our role to be honest”  “I guess they [people with SPMI] just don’t really know or can’t seem to comprehend or connect our role I suppose and they just lack insight into what we are actually doing” | Andrew et al. (2019)  Physiotherapists' views about providing physiotherapy services to people with severe and persistent mental illness: a mixed methods study |
|  | "I think that what is special in physiotherapy for patients with anxiety, it’s… that the knowledge that comes from theoretical explanations has to be experienced in the body. By body awareness training, I believe. Making the body conscious. There’s more to it than just talking."  There was this patient who suffered from a lot of anxiety in the evenings. He learned to use some body awareness exercises that we had trained together. And then he could calm himself, he focused on something else. That’s one way to use it. He could of course take a pill of Stesolid [a sedative] instead, but it seems like the exercises are better for him. They become his inner resources, in a way. | Danielsson et al. (2013)  To sense and make sense of anxiety: Physiotherapists' perceptions of their treatment for patients with generalized anxiety |
|  | “Exercise not only promotes physical fitness. but also … improves cardio respiratory function and mental fitness preventing physical disorders that could develop from a sedentary lifestyle.”  “I have personally seen how physical activity can increase individuals’ energy level, attention to task, focus, decrease anxiety, decrease weight, improve self-esteem, and increase socialization”  “Physio’s are physical health specialists, whilst most others are mental health specialists and do not understand the physical nature of schizophrenia. Therefore, we have to take a lead as it is not safe for all patients to jump in at the deep end, often we have to develop graded exercise programmes.” | Stubbs et al. (2014)  The assessment, benefits and delivery of physical activity in people with schizophrenia: a survey of members of the International Organization of Physical Therapists in Mental Health |
|  | ‘‘Physiotherapists are integral for treating schizophrenia. With the grossly reduced physical health of this patient group we have to take a lead as we are often the leading experts on physical health in the MDT, others are mental health specialists predominantly’’  ‘‘The role of [the] Physiotherapist is important because they can help patients use the body to help the mind to be better’’  ‘‘Physiotherapists help individuals with schizophrenia to socialize, improve cognitive and motor skills, face the reality, [and] experience success through the physical activity. Physiotherapists help also patients to get more self-confidence and self-awareness’’ | Stubbs et al. (2014)  Understanding the role of physiotherapists in schizophrenia: an international perspective from members of the International Organisation of Physical Therapists in Mental Health (IOPTMH). |
|  | “… you have to be careful, I suppose that you don’t sort of feel like you’re going into an area that you’re not really qualified in.” | Tehrany et al. (2024)  Allied health professionals’ experiences and views towards improving musculoskeletal services in the UK for patients with musculoskeletal and co-existing mental health conditions: a qualitative study. |
| Theme 3: Physiotherapists’ Encounters with Multifactorial Psychological Distress | “My lymphedema clients can be incredibly complex with the other stuff, their lymphedema might be incredibly simple, and easy to manage. But the other health concerns, social concerns, functional concerns can be really complex…I’m not saying that other client groups don’t come with complexities but I definitely would not expect that people [with more acute health issues] have the breadth of complex mental health stresses [that I see in lymphedema care]..They often don’t realize how complex or how emotionally draining they can be”.  “A lot of our patients . . ., are coming from a history of abuse and trauma. In my practice, I have heard a lot of stories of sexual abuse and domestic violence” (Lucy)  “I’ve got a few cases in mind of people who are like in distress . . . linked to a domestic violence [situation]” (Mary) | McGrath et al. (2024a)  “People suffer and we see this”: a qualitative study of the forms of patient psychological distress encountered by physiotherapists. |
|  | “It’s so common in my role [as a neurological physiotherapist]. You have some patients who are really low in mood, [and] really hard to engage . . . We often see stress and psychological issues with both the patient themselves and the family member who’s supporting them” (Peter)  “In my experiences, how patients have exhibited psychological distress [is] quite varied. Sometimes . . . those experiencing psychological distress were often withdrawn from care, very difficult to engage . . ., very unmotivated . . . Some I noticed when [receiving] difficult news would shake or have difficulty just keeping eye contact . . . Others I knew would turn to booze or smoking or drugs, not often [would they tell] us this, but the signs were there” (Caroline) | McGrath et al. (2024b). “We take on people’s emotions”: a qualitative study of physiotherapists’ experiences with patients experiencing psychological distress |
|  | “For sure this [the bodily symptoms the patient suffers from] is about the trauma, it is all stored. – (SP): How do you think it is stored? – In the body, the traumatic experiences”.  “She is not really there in the world. That´s the hypothesis when I see her, which is maybe not ‘classic’ physiotherapeutic (laughs), but that´s what I think about it. She is very cautious, maybe she was hurt quite much, maybe she has the feeling she cannot trust anyone, and she has no strength in herself, no life energy”. | Perner, S. and Danielsson, L., 2022. Exploring clinical reasoning in Austrian mental health physiotherapy: the physiotherapist´ s perspective. |
| Theme 4: Psychological Strain Experienced by Physiotherapists | “It’s one of those things where spending energy on empathizing with somebody is exhausting…You try not to take on too much of their concerns and their stress and most of the things but inevitably, it will happen when you will have a day of particularly heavy or particularly crushing, sad stories that you’ve heard”.  “I do not have PTSD. I’ve never [experienced the situation that my patient has experienced but] I’m distressed, just by the nature of empathy in the human condition. This freaks me out” (Lynn)  “We take on people’s emotions . . . So sometimes . . . I can just feel myself choking up, you know, and I think, oh, God, this is just awful . . . I kind of try and kind of quickly check myself and be like, but actually, this is not about me” (Beth)  “If you’re not careful. I actually do think there can be an accumulative effect of listening to lots of people’s difficult stories” (Beverley) | McGrath et al. (2024b) “We take on people’s emotions”: a qualitative study of physiotherapists’ experiences with patients experiencing psychological distress |
|  | “After having one or more of those patients that day, I feel that it does something to me. I get very depressed myself. It’s hard” | Lundin & Bergenheim (2020) Encountering suicide in primary healthcare rehabilitation: the experiences of physiotherapists |
|  | My wife is a [health professional], so occasionally [I debrief] with her. | McGrath et al. (2020)  Trust, conversations and the ‘middle space’: A qualitative exploration of the experiences of physiotherapists with clients with suicidal thoughts and behaviours |
|  | “I found it massively overwhelming. And I wonder… if that could have been a contributing factor to me leaving the NHS… not just depressed people, but I think I found working with people and all their emotions all day just too much actually.”  “You have those patients that you know, [who] are draining and mental health patients often are quite draining, to be honest with you ‘cause they need more, they need more support and motivation.” | Tehrany et al. (2024)  Allied health professionals’ experiences and views towards improving musculoskeletal services in the UK for patients with musculoskeletal and co-existing mental health conditions: a qualitative study. |
| Theme 5: Multilevel Barriers to Integrating Physiotherapy in Mental Health Care | "it’s difficult for them to undergo the activities because of their situation in such they are depending on the medication"  "Patients are more likely to stay in bed and they have to be strongly motivated to be active"  “Many patients can hide behind diagnosis, I am too ill to exercise. Some patients can play up to avoid it” | Soundy et al. (2014)  Barriers to and facilitators of physical activity among persons with schizophrenia: a survey of physical therapists |
|  | “We don’t know where we could send them and we don’t ever get asked to refer them anywhere” | Andrew et al. (2019)  Physiotherapists' views about providing physiotherapy services to people with severe and persistent mental illness: a mixed methods study |
|  | “I don’t know how to refer a patient who is undiagnosed with a mental illness. I don’t know when to refer to a psychologist or psychiatrist” | Hooblaul et al. (2020)  The knowledge, attitudes and perceptions of physiotherapists in KwaZulu-Natal, South Africa, towards mental health |
|  | “We have such high demands on us to perform and accomplish a great deal during our days. You are very focused on managing your day, really. That means you might not be able to settle down or reflect that much afterwards. So, the time pressure I think is … or the pressure in general I think is a factor that makes it difficult”  “I see a problem in that I don’t really know where to turn. What if it is really urgent – where do I turn? That would be interesting to talk or learn more about”  “It is not always easy to get hold of a psychologist…or whoever the patient may need. Instead, the patient will have to wait for three months before seeing someone, and then it might already be too late” | Lundin & Bergenheim (2020) Encountering suicide in primary healthcare rehabilitation: the experiences of physiotherapists |
|  | “… so we haven’t got time to address it, but the other part is you have to because otherwise, they’re not going to get better…”  “[…]there’s a massive shortage in clinical psychologists…so I think that’s the problem.”  “It wasn’t provided by our service, but we sort of linked up with them. And I think their service was underfunded.”  “yeah, definitely. The time is the barrier, at the moment. So something has got to give in order to do that.“ | Tehrany et al. (2024)  Allied health professionals’ experiences and views towards improving musculoskeletal services in the UK for patients with musculoskeletal and co-existing mental health conditions: a qualitative study. |
| Theme 6: Enablers to Engaging in Physiotherapy Services | “A good discussion around what exercise the individual is interested in and tailoring a programme around this”  “Increase the accessibility of physical activity”  “Supervised sessions”  “support from the treatment team; a well trained staff that makes the patient feel  safe” | Soundy et al. (2014)  Barriers to and facilitators of physical activity among persons with schizophrenia: a survey of physical therapists |
|  | “It's all about establishing that trust” | McGrath et al. (2020)  Trust, conversations and the ‘middle space’: A qualitative exploration of the experiences of physiotherapists with clients with suicidal thoughts and behaviours |
|  | “I think we can reduce the isolation when we walk around the courtyard with the patient” | Moll et al (2021)  Physiotherapists and Psychiatric Hospitalization in General Hospitals: Care and Professional Training. |
|  | “So that’s a thing you have to manage the person and then if you work in other environments, you’re also managing their family. It’s not just the person. It’s their families’ mental health problems that are influencing the way your patient is. And so, you know, it can just become really complex.”  “I think one of the things that’s probably really important is supervision. Having that space to be able to reflect on your caseload, and when those people have come up with mental health problems or you’re concerned about that is having someone and that kind of opened up a can of worms and they won’t be qualified and they don’t know what to do and how to deal with it.” | Tehrany et al. (2024)  Allied health professionals’ experiences and views towards improving musculoskeletal services in the UK for patients with musculoskeletal and co-existing mental health conditions: a qualitative study. |

**Student**

| Themes | Participants quotations from primary studies | Reference |
| --- | --- | --- |
| Theme 1: Perceived Gaps in Education and Training | “[Individuals with a serious mental illness] are definitely a population I hadn’t really thought about specifically. It’s not something that we really learn a lot about in physical therapy.” | Zechner et al (2022)  Physical therapy students’ perceptions for working with persons with mental illness in the USA |
|  | “I don’t feel like it, in terms of disclosure of self-harm specifically, I don’t feel like there’s been anything that I’ve learnt at University that I feel would help or that has developed my communication with such patients”  “if someone told me anything like that [disclosed intention to self-harm], I don’t feel confident to intervene, so I would just refer to the mental health physios or, say that if it’s in the hospital setting, let the other staff know; that’s all I would do. I don’t know about the procedures.”  “I’ve never seen or been made aware of even in sort of like placement handbooks things such as policies and stuff relating to disclosure of self-harm or intent to self-harm, which would have been quite a handy thing to know. I think there’s more potential for it in a couple of the placements I’ve been on. Yeah, I think policy wise it is like a safeguarding thing, and that would be my only way I would know - other than like talking to my line manager or my educator whilst on placement about the situation” | Gamblin et al (2024)  “I haven't got a clue what policies are in place for this!”: physiotherapy students’ preparedness to deal with self-harm. |
|  | “We did psychology in the first year, but we did not learn any practical skills; if a patient has a certain condition, what I can do for them” (Participant 1, Focus group 2, 2nd year student)  “I need to know a lot and everything there is to know about mental health.” (Participant 2, Focus group 2, 4th year student) | Hooblaul et al. (2024)  Physiotherapy students’ and officers’ knowledge, attitudes, and perceptions of mental health |
|  | “I don’t feel that competent to be kind of managing their, like the approach to mental health within physiotherapy practice.” | Byrd et al. (2025).  Exploring the Confidence and Attitudes of Physiotherapy Students Working with People Experiencing Mental Ill Health: A Mixed-Methods Study |
| Theme 2: Value of Practical and Experiential Learning | “If I had not experienced my supervisor pushing me to step outside of my comfort zone, I would have missed a lot of learning, I think. I learned a lot from asking difficult questions. It is unpleasant to talk about the issues that are a bit taboo, in a way. But you learn a lot about yourself too; how to react when being challenged.” | Tessem et al. (2022)  Learning from a situation of discomfort–a qualitative study of physiotherapy student practice in mental health |
|  | “The most important thing I took away…[it] was just a joyful experience. I really enjoyed it. I thought that it was something that was interesting. I really enjoyed participating in it. It was beneficial to me as a student to prepare me for my career and I thought that it was beneficial to them just to get [the people with SMI] up and moving…[with] an hour of something fun to do.” | Zechner et al (2022)  Physical therapy students’ perceptions for working with persons with mental illness in the USA |
|  | “BBAT could be useful for body and mental relaxation”  “BBAT had future due to demonstrate improvements and the experience has been good” | Bravo et al. (2022)  Meeting current needs in mental health physical therapy: A qualitative study of students’ experiences |
|  | “I think just having more patience with people… knowing that people may be in a very different life situation”  “Having a greater understanding of the population and again feeling much more comfortable with working with them, because maybe if had I been a new grad going out there and on my first affiliation, I’d get someone who was listed as having a severe mental illness and I might have been a little bit taken back or a little bit okay I’m not sure what to expect, what to do and I think it did definitely help me with that comfort level” | Karyczak et al (2020)  Service learning experiences of doctor of physical therapy students with a severe mental illness population |
|  | Be patient, listen more, don’t just be there to provide physiotherapy, sometimes you need to read patient notes to identify whether they have mental health problems (responses to the open-ended question) | Edgar & Connaughton (2021)  Using mental health first aid training to improve the mental health literacy of physiotherapy students |
| Theme 3: Strategies to Improve Confidence and Attitudes of Students in Mental Health | “So, I think having exposure to that (Pt with MI) would definitely increase confidence especially with guidance from a mental health physio who has a lot of knowledge in that area” | Byrd et al. (2025).  Exploring the Confidence and Attitudes of Physiotherapy Students Working with People Experiencing Mental Ill Health: A Mixed-Methods Study |
|  | “I’m not as judgmental as otherwise I would be. It definitely prepared me (better) than other people who are just used to the regular outpatient population… People with a severe mental illness can still exercise and participate in society and they want to and they enjoy that” | Karyczak et al. (2020)  Service learning experiences of doctor of physical therapy students with a severe mental illness population |
|  | “I believed that BAs (body awareness) was so far field from PT, but now I change my mind” | Bravo et al. (2022)  Meeting current needs in mental health physical therapy: A qualitative study of students’ experiences |
| Theme 4: Challenging Experiences of Working with People with Mental Illness as a Catalyst for Learning | “She was running on a treadmill, and suddenly she just [mentally] disappeared completely. She was running slower and slower, and I couldn’t get in touch with her. It was scary, but I learned a lot from it.” | Tessem et al. (2022)  Learning from a situation of discomfort–a qualitative study of physiotherapy student practice in mental health |
|  | “We came up with how we thought we would approach the physical activity portion and within the first week quickly realized that what we had planned was not going to work …[we] had to change our approach. I think that was very helpful. Going in with this plan and then reading your patients, and then getting an idea of what works for them and having to change [the plan] to meet their needs.” | Zechner et al (2022)  Physical therapy students’ perceptions for working with persons with mental illness in the USA |
|  | “Adapting your exercises based on each patient, individually tailoring things to keep up their confidence. Because sometimes you try to get your patient to do something ‑ it might be a little too challenging and you see them kinda like knock them down or they kinda get discouraged or if they have a low frustration level” | Karyczak et al (2020)  Service learning experiences of doctor of physical therapy students with a severe mental illness population |
